# COVID-19-Associated Sepsis: Potential Role of Phytochemicals as Functional Foods and Nutraceuticals

**DOI:** 10.3390/ijms25158481

**Published:** 2024-08-03

**Authors:** Bruno de Souza Goncalves, Darshan Sangani, Aleen Nayyar, Raghav Puri, Mahir Irtiza, Asma Nayyar, Abdelnaby Khalyfa, Komal Sodhi, Sneha S. Pillai

**Affiliations:** 1 Department of Surgery, Internal Medicine and Biomedical Sciences, Joan C. Edwards School of Medicine, Marshall University, Huntington, WV 25701, USA; desouzagonca@marshall.edu (B.d.S.G.); sangani4@marshall.edu (D.S.); puri1@marshall.edu (R.P.); mahirirtiza07@gmail.com (M.I.); nayyara@marshall.edu (A.N.); khalyfa@marshall.edu (A.K.); sodhi@marshall.edu (K.S.); 2 Department of Medicine, Sharif Medical and Dental College, Lahore 55150, Pakistan; aleen.nayyar@gmail.com

**Keywords:** COVID-19, sepsis, phytochemicals, functional foods, nutraceuticals

## Abstract

The acute manifestations of coronavirus disease 2019 (COVID-19) exhibit the hallmarks of sepsis-associated complications that reflect multiple organ failure. The inflammatory cytokine storm accompanied by an imbalance in the pro-inflammatory and anti-inflammatory host response to severe acute respiratory syndrome coronavirus 2 (SARS-CoV-2) infection leads to severe and critical septic shock. The sepsis signature in severely afflicted COVID-19 patients includes cellular reprogramming and organ dysfunction that leads to high mortality rates, emphasizing the importance of improved clinical care and advanced therapeutic interventions for sepsis associated with COVID-19. Phytochemicals of functional foods and nutraceutical importance have an incredible impact on the healthcare system, which includes the prevention and/or treatment of chronic diseases. Hence, in the present review, we aim to explore the pathogenesis of sepsis associated with COVID-19 that disrupts the physiological homeostasis of the body, resulting in severe organ damage. Furthermore, we have summarized the diverse pharmacological properties of some potent phytochemicals, which can be used as functional foods as well as nutraceuticals against sepsis-associated complications of SARS-CoV-2 infection. The phytochemicals explored in this article include quercetin, curcumin, luteolin, apigenin, resveratrol, and naringenin, which are the major phytoconstituents of our daily food intake. We have compiled the findings from various studies, including clinical trials in humans, to explore more into the therapeutic potential of each phytochemical against sepsis and COVID-19, which highlights their possible importance in sepsis-associated COVID-19 pathogenesis. We conclude that our review will open a new research avenue for exploring phytochemical-derived therapeutic agents for preventing or treating the life-threatening complications of sepsis associated with COVID-19.

## 1. Introduction

The coronavirus disease 2019 (COVID-19) has rapidly disseminated worldwide, impacting millions of people [1]. COVID-19 exhibits a broad spectrum of clinical manifestations, ranging from mild respiratory symptoms to severe pneumonia and acute respiratory distress syndrome (ARDS) [2]. COVID-19-associated sepsis has become a significant concern among ARDS patients [3]. Sepsis, characterized as life-threatening organ dysfunction resulting from a dysregulated host response to infection, disrupts the normal function of the immune, respiratory, cardiovascular, renal, and central nervous systems, thereby disturbing metabolic homeostasis and increasing the morbidity and mortality linked with COVID-19 [4,5]. The intricate interaction between the viral infection and the host immune response triggers a cytokine storm, a hyper-inflammatory condition that substantially contributes to sepsis pathogenesis in COVID-19 [6,7,8]. This scenario underscores the need to explore adjunctive therapeutic strategies to mitigate the inflammatory response and improve patient outcomes.

Phytochemicals are bioactive compounds found in plants, and they have been recognized for their anti-inflammatory, antioxidant, and immunomodulatory properties [9,10,11,12,13]. Investigating phytochemicals as potential therapeutic agents against COVID-19-associated sepsis is an emerging and promising field. Compounds such as flavonoids and phenolic acids have demonstrated potential in modulating inflammation and reducing oxidative stress, which is critical in the context of sepsis [14]. As functional foods and nutraceuticals, phytochemicals provide the dual benefits of nutritional value and therapeutic potential, presenting a viable adjunctive strategy to conventional medical treatments.

Given the importance of identifying effective interventions for COVID-19-associated sepsis, the incorporation of phytochemicals as functional foods and nutraceuticals offers a promising pathway. The central objective of this review article is to provide a comprehensive overview of the current understanding of COVID-19-associated sepsis, its underlying pathophysiological mechanisms, and the potential therapeutic benefits of phytochemicals. Briefly, the pathogenesis of COVID-19-associated sepsis was described in the first section, which gives the information about the mechanism of disease progression and multi-organ damage. The current treatment approaches for COVID-19-associated sepsis and its challenges are included in the next section to highlight the need for alternative therapeutic interventions. The importance of advanced research on phytochemicals as nutraceuticals and functional foods is also briefed, followed by a detailed description of six potent phytochemicals with promising pharmacological properties against sepsis and COVID-19, which are present in the daily diet. Consolidated information on each phytochemical from various research studies, including clinical trials in humans on sepsis and COVID-19, is presented concisely. Additionally, we highlight the challenges and future research directions in this expanding field, emphasizing the necessity for clinical trials to validate the efficacy and safety of these natural compounds.

## 2. Pathogenesis of COVID-19-Associated Sepsis

The pathogenesis of COVID-19-associated sepsis involves a multifaceted interplay between viral replication, host immune response, and subsequent tissue damage [15,16]. A cascade of immune reactions is triggered by severe acute respiratory syndrome coronavirus 2 (SARS-CoV-2) after it enters the host cell via the angiotensin-converting enzyme 2 (ACE2) receptor [17,18,19,20]. The initial immune response is characterized by the activation of innate immunity and the release of pro-inflammatory cytokines such as interleukin (IL)-6, tumor necrosis factor-alpha (TNF-α), and IL-1β [21,22,23,24]. This hyper-inflammatory response, often referred to as a cytokine storm, plays a pivotal role in the progression of sepsis, leading to tissue damage and organ dysfunction [25,26,27,28,29,30]. The cytokine storm associated with COVID-19 sepsis leads to endothelial dysfunction, increased vascular permeability, and coagulopathy, contributing to multi-organ failure that includes the lungs, kidneys, liver, and heart [16]. The higher levels of ACE2 in endothelial cells as compared to other cells make them specific targets for viral infection [31]. This endothelial dysfunction exacerbates microvascular thrombosis, which is a hallmark of severe COVID-19 and sepsis [8]. Additionally, the adaptive immune response also plays a significant role in the pathogenesis of COVID-19-associated sepsis [8,32,33]. T-cell lymphopenia, a common finding in severe COVID-19 cases, impairs the host’s ability to mount an effective antiviral response [34,35]. The depletion of CD4+ and CD8+ T-cells and an imbalance in regulatory T-cells and effector T-cells contribute to immune dysregulation [36,37,38]. This dysregulated immune response fails to control viral replication effectively and perpetuates the inflammatory cascade, further exacerbating sepsis.

**Figure 1:**
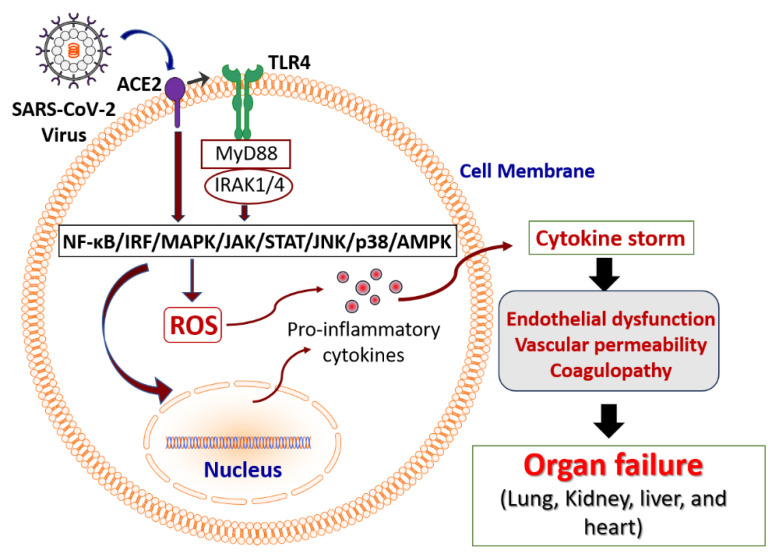
Pathogenesis of COVID-19-associated sepsis: SARS-CoV-2 infection activates various signaling pathways that release pro-inflammatory cytokines, ultimately leading to pathophysiological complications of sepsis that end in life-threatening multi-organ failure. Abbreviations: Severe acute respiratory syndrome coronavirus 2 (SARS-CoV-2), angiotensin-converting enzyme 2 (ACE2), toll-like receptor 4 (TLR4), myeloid differentiation primary response 88 (MYD88), interleukin-1 receptor-associated kinase 1 and 4 (IRAK1/4), nuclear factor kappa B (NF-κB), interferon regulatory factors (IRF), mitogen-activated protein kinase (MAPK), Janus kinase (JAK), signal transducer and activator of transcription (STAT), c-Jun N-terminal kinases (JNK), AMP-activated protein kinase (AMPK), reactive oxygen species (ROS).

COVID-19-associated sepsis exerts a widespread impact on multiple organ systems [15,16]. As the primary viral entry and replication site, the lungs are most frequently and severely affected [25]. The engagement of pattern recognition receptors (PRRs) and toll-like receptors (TLR4) by the virus triggers the nuclear factor-kappa B (NF-κB) and interferon regulatory factor (IRF) pathways, resulting in the production of pro-inflammatory cytokines [39,40]. In addition, the mitogen-activated protein kinase (MAPK) pathway is activated, further amplifying the inflammatory response and inducing apoptosis of alveolar epithelial cells [41]. The pathophysiology extends beyond the pulmonary system, leading to a critical involvement of the kidneys, liver, and cardiovascular system [26,28,30]. The pathological complications associated with COVID-19-associated sepsis are schematically represented in Figure 1.

Acute kidney injury (AKI) is a common complication, driven by a combination of direct viral effects, systemic inflammation, and microvascular thrombosis [30]. The impact on the kidneys occurs through the activation of the NF-κB and IRF pathways, initiating a robust inflammatory response [42]. The Janus kinase/signal transducer and activator of transcription (JAK/STAT) pathway is also implicated, leading to the activation of STAT, a key protein involved in the expression of genes related to inflammation and apoptosis [42]. Hepatic dysfunction, manifesting as elevated liver enzymes and liver failure, is also prevalent, indicative of hepatocellular injury and systemic inflammatory response [22,43,44]. The recognition of viral components by PRRs on hepatic cells activates downstream signaling pathways, including the NF-κB, MAPK pathway, particularly the c-Jun N-terminal kinase (JNK) and p38 MAPK, hypoxia-inducible factor-1 alpha (HIF-1α), and JAK/STAT signaling pathway, promoting the expression of inflammatory mediators and inducing hepatocyte apoptosis and fibrosis [45,46]. Cardiovascular complications such as myocarditis, heart failure, and thromboembolic events are frequently observed, exacerbated by the pro-thrombotic state induced by the virus and inflammatory processes [26,47]. The impact on the heart occurs through the activation of inflammatory and stress-signaling pathways that mediate cardiomyocyte apoptosis and fibrosis [48]. The neurological system is also affected, with complications ranging from encephalopathy to cerebrovascular events [49,50]. The multi-organ dysfunction observed in COVID-19-associated sepsis underscores the complexity of the disease and the need for a comprehensive understanding of its pathogenesis to improve patient management and outcomes.

## 3. Current Treatment Approaches for COVID-19-Associated Sepsis and the Challenges

COVID-19-associated sepsis presents unique clinical challenges, necessitating tailored therapeutic strategies to improve patient outcomes [15]. Various treatment strategies are being investigated, including drug-related therapies such as corticosteroids, immunotherapy, and anticoagulant therapy [51,52,53,54,55]. Corticosteroids, a group of steroid hormones, are utilized for their anti-inflammatory properties that function by suppressing the cytokine storm [56,57,58]. These steroids bind to cytosolic glucocorticoid receptors (GRs), which then translocate to the nucleus and reduce gene transcription by interacting with pro-inflammatory transcription factors such as activation protein-1 (AP-1) and NF-kB [59]. It is indicated that corticosteroids can improve the quality of life for hospitalized COVID-19 patients with sepsis [60]. Dexamethasone, a glucocorticoid, has shown effectiveness in reducing mortality among severe COVID-19 patients requiring oxygen support [51]. A similar study showed that the early administration of dexamethasone reduced the duration of mechanical ventilation and overall mortality in patients with moderate-to-severe ARDS [54]. Dexamethasone therapy also reduced the severity of inflammation by severe cytokine storm inhibition in COVID-19-related pneumonia patients [61]. Additionally, dexamethasone decreased plasma biomarkers of lung epithelial/endothelial injury and inflammation, demonstrating that the positive effect of dexamethasone in ameliorating severe COVID-19 may be related to pathways of inhibition of epithelial and inflammatory damage [62]. Methylprednisolone is another corticosteroid that has shown effectiveness in severe and critical COVID-19 patients [63]. This suggests that methylprednisolone is an efficient therapeutic agent for hospitalized severe COVID-19 patients in the pulmonary phase [63]. Another study revealed that early administration of low-dose methylprednisolone significantly decreased death rates and reduced ventilator dependence in patients with severe COVID-19 pneumonia [56]. Additionally, methylprednisolone treatment ameliorated levels of myoglobin and inflammatory response, decreasing monokine-induced Interferon-gamma (IFN-γ) and IFN-γ-induced Protein-10 levels in hospitalized COVID-19 patients [64].

Immunotherapy employs agents that enhance or suppress the immune response [65]. In COVID-19-associated sepsis, an overactive immune response can lead to excessive inflammation and tissue damage, characteristic of cytokine storms [15]. Anakinra, a recombinant IL-1 receptor antagonist, blocks the activity of the pro-inflammatory cytokines IL-1α and IL-1β, preventing sterile inflammation and inflammasome assembly, and it has been associated with lower mortality rates in COVID-19 patients [55]. A similar study showed that Anakinra was effective in reducing the clinical signs of hyperinflammation in critically ill COVID-19 patients [66]. Additionally, Anakinra reduced the need for invasive mechanical ventilation in the intensive care unit (ICU) and mortality among patients with severe forms of COVID-19 [67]. Tocilizumab is an anti-IL-6 receptor antibody that significantly affects the treatment of infection-induced cytokine storm [68]. It was shown that earlier use of tocilizumab in COVID-19 patients was beneficial for survival, hospitalization length, and oxygen support duration [69]. Another study revealed that tocilizumab reduced short-term mortality, intensive care unit admission, serious infection, serious adverse events, the chances of requiring invasive mechanical ventilation, and time-to-hospital discharge in hospitalized COVID-19 patients [70,71]. Another type of therapy is the use of anticoagulants, which are being developed to address thrombosis, a common complication in severe COVID-19 cases [53]. Anticoagulation with low-molecular-weight heparin (LMWH) or direct oral anticoagulants (DOACs) is often employed to reduce the risk of thrombotic events, which can exacerbate sepsis [72,73].

The treatment of COVID-19-associated sepsis has numerous challenges that complicate the effective management of this severe complication. One primary challenge is the heterogeneity of sepsis presentations among COVID-19 patients, which complicates the development of standardized treatment protocols and necessitates individualized therapeutic approaches [74,75]. The rapid progression from mild COVID-19 symptoms to severe demands timely and often aggressive intervention, a particularly challenging requirement in overburdened healthcare systems [76,77]. Additionally, the use of therapies such as corticosteroids and immunomodulators, while beneficial, also carries risks of secondary infections and other adverse effects, necessitating careful risk–benefit analyses [78]. Despite this, all therapeutic options require further study due to the absence of an effective treatment. While significant strides have been made in understanding and managing this complex condition, ongoing research and adaptive clinical strategies are essential to address the evolving challenges presented by this global health threat.

## 4. Importance of Phytochemicals as Functional Foods and Nutraceuticals

Since ancient times, the therapeutic importance of herbal medicine has been an essential component of the cultures and traditions of various countries to treat diseases [79]. Phytochemicals are plant-derived bioactive compounds produced for their protective effects. Phytochemicals provide healthcare defense to the human body by virtue of their properties, such as antioxidant, anti-inflammatory, antimicrobial, antidiarrheal, anthelmintic, antiallergic, antispasmodic, anti-obesity, antihypertensive, antiviral, antidiabetic, and anti-cancer properties [80,81,82,83,84,85,86,87]. Medicine derived from phytochemicals has shown fewer complications than synthetic counterparts, as they are more affordable, less toxic, and have fewer side effects [88,89,90]. Phytochemicals are reported to act as either functional foods to protect against diseases or as nutraceuticals that are complementary drugs to treat disease pathology with fewer side effects [91,92]. Functional foods normally provide health benefits by optimizing the physiological system to prevent or control chronic diseases in addition to their basic nutrient supply [93]. Nutraceuticals can be considered the purified form of active phytochemicals that can treat one or more chronic diseases in a pharmaceutical form [94,95,96,97]. Hence, phytochemicals can be considered as potential candidates for the management of chronic diseases, which warrants further research to explore the detailed prevention/treatment modality in each disease.

Recent studies have shown that phytochemicals can be used as a method to prevent chronic and degenerative diseases such as cancer, cardiovascular disease, dementia, diabetes, etc. [98,99,100,101]. They are reported to have anti-cancer and anti-inflammatory properties in various cancer cells, resulting in cell cycle arrest and apoptosis. [102,103]. Clinical studies have shown that the long-term consumption of phytochemicals may attenuate several neuropathological conditions associated with the development of Alzheimer’s disease [104]. Plant-derived chemicals have properties that lower adipose tissue, modulate lipid and carbohydrate metabolism, and exert antioxidant and anti-inflammatory properties that together lower the risk of obesity [105]. Phytochemicals can release nutritional signals that reverse neuroinflammation and that, in turn, provide neuroprotective abilities against neurodegenerative diseases [106]. Phytochemicals modulate transcription factors related to cholesterol metabolism, resulting in lessening the chances of liver and heart diseases [107,108,109]. The therapeutic efficacy of various phytochemicals has been demonstrated to alleviate sepsis and its life-threatening complications. [110,111,112,113]. The protective effects exerted by these phytochemicals can be attributed to their potent antioxidant and anti-inflammatory properties that modulate the inflammatory response and biochemical pathways associated with sepsis. Recently, several studies have shown that therapeutic plants containing phytochemicals are efficient in treating inflammatory-related diseases initially caused by SARS-CoV2 [114,115,116,117,118]. Hence, in this review, we aim to highlight the importance of some potent phytochemicals with remarkable therapeutic potential against sepsis and COVID-19, which will be a guide for their possible future research applications in sepsis associated with SARS-CoV-2 infection. Table 1 and Table 2 summarize the findings.

## 5. Importance of Phytochemicals as Potential Agents for COVID-19-Associated Sepsis

In the present review, we focus on six potent phytochemicals with promising pharmacological actions, which are commonly present in daily diets. In the following sections, we consolidate the findings from various studies, including clinical trials in humans, to explore more into the therapeutic potential of each phytochemical against sepsis and COVID-19.

### 5.1. Quercetin

Quercetin is a flavonoid molecule found in everyday fruits and vegetables, especially in citrus fruits, broccoli, cherries, berries, grapes, and onions [241]. The abundance of quercetin differs by food group, with certain foods like onion having an extensive amount. However, the abundance of quercetin does not correlate with its bioavailability in the body once consumed. After intestinal absorption and phase I and II metabolism, quercetin undergoes a series of metabolic reactions in the liver, and the resulting metabolites are released into the circulation and excreted into urine through the kidney [242]. In the liver, quercetin undergoes metabolism to sulfate, glucuronide, and methyl groups, which can be then assessed for bioavailability by their concentration in blood and urine [242,243]. Through this process, however, quercetin has been shown to have a low bioavailability and thus low bioactivity in the body [244,245]. Fortunately, new formulations of quercetin, such as the Quercetin Phytosome delivery system, have been shown to improve the bioavailability of quercetin, increasing the possibility of extracting the positive effects of the phytochemical [246]. Quercetin has been shown to exhibit a wide range of properties that apply to clinical medicine [247]. Through various studies, quercetin has demonstrated broad-spectrum antibacterial, antioxidant, antiviral, and anti-inflammatory properties [225,248,249,250,251,252]. In addition, studies have exhibited the potency of quercetin against cardiovascular diseases, carcinogenesis, and neurotoxic effects of mycotoxins, along with its immunomodulatory effects [253,254,255,256,257,258,259]. Furthermore, a randomized controlled clinical trial study in humans demonstrated that quercetin decreased plasma LDL and systolic blood pressures in overweight subjects, highlighting its anti-obesity properties [254]. Also, it was beneficial in fighting neurodegenerative diseases when combined with fish oil [260]. These characteristics suggest that quercetin is a potent nutraceutical whose use in specific disease states is a potential avenue for research.

Quercetin has exhibited a unique potential role in managing sepsis [261]. In lipopolysaccharide-induced (LPS) sepsis models of in vitro and in vivo studies, quercetin has been shown to reduce the phosphorylation and degradation of the inhibitor of κBα (IκBα), thus downregulating the activity of NF-κB and causing an overall marked decrease in systemic and macrophage production of TNF-β and IL-1β [119]. Quercetin has not only demonstrated beneficial effects in fighting systemic inflammation created by sepsis but has also shown promise in preventing/treating sepsis-induced specific organ damage. In a septic rat model, quercetin demonstrated the inhibition of reactive oxygen species (ROS) in the lung tissue and increased the expression and activity of ROS-scavenging enzymes like superoxide dismutase (SOD), catalase (CAT), and ascorbate peroxidase (APX) [120]. It also decreased the levels of high-mobility group box 1 (HMGB1) protein expression, which has been shown to play an inflammatory role in sepsis [120,121]. Furthermore, quercetin could suppress the nuclear translocation of NF-κB and reduce inflammatory cytokine levels by modulating the NADPH oxidase-2 (NOX2)/ROS/NF-kB pathway in LPS-treated lung epithelial cells [122].

Quercetin has been shown to attenuate sepsis-induced stress on the endoplasmic reticulum and mitochondria, thus possibly preventing acute lung injury (ALI) [123]. Specifically, quercetin has demonstrated the activation of the SIRT1/AMP-activated protein kinase (AMPK) pathway, which protects the ER and mitochondria of lung alveolar epithelial cells, thus reducing the likelihood of ALI [123]. Also, quercetin has been shown to be preventive against sepsis-induced ALI via the inhibition of cytokine production, decreasing inflammatory cell recruitment, and reducing the expression of inflammatory enzymes [124]. In addition, quercetin has been shown to mitigate sepsis-induced ARDS, as it blocks the effects of NOD-like receptors, NF-κB, TNF, and HIF-1, thus reducing overall oxidative stress, inflammation, and tissue damage [125]. Rutin, a combination of quercetin and rutinose (disaccharide), has shown efficacy as a possible prophylaxis for sepsis-induced cardiomyopathy and cardiac apoptosis, according to cecal ligation and puncture (CLP) surgery mouse models [126]. The specific outcomes noted were an overall decrease in pro-inflammatory cytokines, such as TNF-α, IL-6, cardiac troponin T (cTNT), and an increase in IL-10 when the mice were pretreated with rutin [126]. Quercetin has been shown to diminish levels of NOX2/ROS-mediated NF-kB/thioredoxin-interacting protein (TXNIP) pathway, thus preventing mitochondrial damage to cardiomyocytes experiencing sepsis-induced pyroptosis in CLP rat models [127]. Furthermore, Tamarixetin, is a derivative of quercetin, and it has shown anti-inflammatory effects during bacterial sepsis mouse models via increased production of IL-10-secreting cells in the spleen and thus an increased production of anti-inflammatory cytokine IL-10 [128]. In addition, quercetin is a component of the Chinese herbal extract Xuebijing, and it has been shown to regulate C-X-C Motif Chemokine Ligand 8 (CXCL8), thus posing as a potential avenue for treatment against sepsis-associated kidney injury [129]. These extensive mechanism findings suggest that quercetin plays a beneficial role in fighting sepsis and could thus be utilized in patient management as a supplementation to standard care.

Quercetin has shown a multitude of anti-SARS-CoV-2 effects through multiple studies [262]. Quercetin has demonstrated inhibitory activity against the highly conserved protease 3 chymotrypsin-like protease (3CLpro), or Main Proteinase (Mpro), protease enzymes of COVID-19 that are responsible for creating functional proteins for the virus, like RNA-dependent RNA polymerase (RdRp), and it plays a significant role in its replication cycle [190]. According to network pharmacology and molecular docking studies, quercetin has exhibited the ability to bind to the active sites of both 3CLpro via interactions with various amino acids and ACE2, thus possibly posing as a potential therapy for COVID-19-induced AKI [191]. Furthermore, according to Kyoto Encyclopedia of Genes and Genomes (KEGG) pathway analysis, it is proposed that quercetin may treat COVID-19-induced AKI through various pathway interactions such as TLR, HIF-1α, vascular endothelial growth factor (VEGF), TNF, and apoptosis [191]. In addition, quercetin has been shown to disrupt syncytium formation between the spike (S) protein of SARS-CoV-2 and ACE2, thus inhibiting SARS-CoV-2 replication [192]. Quercetin has not only demonstrated anti-COVID-19 activity, but specific formulations have also proven effective in fighting the virus. For example, quercetin in combination with dasatinib has exhibited selectivity in eliminating virus-induced senescence cells triggered by COVID-19, as well as ameliorating inflammation and reminiscent lung disease in SARS-CoV-2-infected hamsters and mice [263]. In addition, nanoparticles incorporated with quercetin and an ACE2 membrane have been shown to decrease the expression of AXL tyrosine kinase receptor, an enzyme that plays a role in SARS-CoV-2 entry into cells [194]. The Huashi Baidu decoction, a traditional Chinese medicine, is composed of 343 different compounds, 6 of which, including quercetin, have demonstrated anti-SARS-CoV-2 effects [264]. These authors reported that quercetin exhibited inhibitory activity against phosphodiesterase type 4 (PDE4) (the agent responsible for neutrophil activation/response in COVID-19) and moderate inhibitory activity against SARS-CoV-2 Mpro, an antiviral drug target [264].

Clinical trials for quercetin and COVID-19 are still in progress, but some have published results [265]. For example, in trials of quercetin in patients with mild COVID-19, it was shown that the phytochemical, when administered as Quercetin Phytosome, increased its bioavailability, decreased symptom severity and incidence, increased viral clearance, and ameliorated COVID-19 biomarkers [196]. Another randomized clinical trial showed the speedy clearance of SARS-CoV-2-associated complications and the modulation of inflammatory responses in patients who received oral quercetin supplements [197]. Furthermore, in a randomized controlled trial of severely hospitalized patients with COVID-19, quercetin, in combination with remdesivir and favipiravir, was associated with earlier hospital discharge and decreased serum levels of alkaline phosphatase (ALP), q-C-reactive protein (q–CRP), and lactate dehydrogenase (LDH), which are all inflammatory markers related to the disease course of COVID-19 [198], thus marking an association with improved disease outcomes. Also, in an exploratory randomized clinical trial utilizing a combination of quercetin and curcumin, another phytochemical, with standard care, it was found that the combination was associated with earlier viral clearance and improved resolution of COVID-19 acute symptoms in patients with early/mild forms of COVID-19 infection [199]. Clinical studies have demonstrated quercetin as not only a treatment for COVID-19 infection but also as a possible protectant against acquiring SARS-CoV-2 [200]. Specifically, according to a 3-month clinical pilot study, it was found that by administering daily Quercetin Phytosome supplementation, patients experienced a protection factor of 14% more than those who did not receive supplementation [200]. Altogether, quercetin has demonstrated an array of properties combatting COVID-19, from specific anti-SARS-CoV-2 activity to preventive efforts, thus serving as a viable therapeutic regimen.

### 5.2. Curcumin

Curcumin is the active component of turmeric, a rhizome of Curcuma longa spice, which has long been recognized for its use in traditional medicine as well as for culinary purposes [266,267]. Curcumin’s pathway in the body starts with absorption, specifically through its metabolites [268]. The phytochemical undergoes extensive metabolism in the gastrointestinal tract and the liver to produce various metabolites including tetrahydrocurcumin and hexahydrocurcumin, which are then conjugated with glucuronic acid and sulfate [269,270]. Curcumin is then predominantly excreted through feces, if administered orally or intraperitoneally, or through the bile, if given intravenously or intraperitoneally [269]. Curcumin’s bioavailability is minuscule when orally administered due to its poor absorption in the gastrointestinal tract and instability in blood pH > 7 [271,272]. To overcome this issue, various efforts have been made to increase the bioavailability of curcumin such as an adjuvant combination with other phytochemicals, nanoparticle-based delivery systems, and liposome and micelle formulations [273,274,275,276,277,278,279]. Curcumin has demonstrated a wide variety of properties applicable to clinical medicine through numerous investigations [272,280,281,282]. Studies have shown that curcumin exhibits anti-cancer, antioxidant, anti-inflammatory, anti-aging, antimicrobial, anti-fungal, and antiviral effects [283,284,285,286,287,288]. Curcumin has also demonstrated protective properties against various disease states including cardiovascular and neurodegenerative diseases, such as Alzheimer’s [289,290,291]. Specifically, clinical trials of curcumin have shown efficacy in battling disease states such as inflammatory-bowel-disease-like ulcerative proctitis (ulcerative colitis affecting the rectum) and Crohn’s disease, rheumatoid arthritis when combined with diclofenac sodium, and it has shown efficacy in delaying pre-diabetic development of type 2 diabetes [292,293,294]. Other clinical trials of curcumin have also shown a decrease in serum lipid peroxidase and cholesterol levels, posing as a possible preventative agent against atherosclerosis [295]. Altogether, the extensive list of clinical applications and promising effects warrants curcumin as a viable candidate for therapy and research.

Through multiple studies, curcumin has exhibited an array of activity against sepsis [296]. An in vivo study showed that curcumin suppressed the mitochondrial signal transducer and activator of transcription (STAT) 3 in macrophages and thus NF-κB activity, therefore attenuating LPS-mediated sepsis [130]. Furthermore, in LPS-induced septic mice, curcumin was shown to enhance the immune function against sepsis through the increase in miRNA-184-5p, utilizing the cathepsin B-mediated phosphatidylinositol 3-kinase (PI3K)/protein kinase B (AKT) pathway [139]. Aside from general systemic anti-inflammatory activity, curcumin has also shown promise in fighting a variety of sepsis-induced disease states. For instance, in a bioinformatic analysis, curcumin was found to ameliorate sepsis-induced cardiomyopathy by blocking TLR1 [132]. Furthermore, in vivo and in vitro studies have also shown curcumin to increase mitochondrial synthesis and prevent destruction via SIRT1-dynamin-related protein 1 (DRP1)/peroxisome proliferator-activated receptor-γ coactivator 1-α (PGC1α) pathway, thus exhibiting promise in treating and protecting against sepsis-induced cardiomyopathy [133]. Curcumin also demonstrated a cardioprotective effect against cardiac lesions in septic mice via modulation of the mammalian target of rapamycin (mTOR) pathway [134]. In in vitro septic rat models, curcumin demonstrated NF-κB and p38 inhibition, resulting in the prevention of sepsis-induced muscle proteolysis [135]. In septic mice models subjected to CLP, it was found that curcumin improved inflammation in the lung and kidneys, decreased levels of TNF-α and IL-6, and upregulated the activity of anti-inflammatory T regulatory cells (Tregs) and the production of IL-10 [136]. In sepsis-induced ALI of CLP rat models, curcumin demonstrated a decrease in inflammation [decreased TNF-α, IL-8, and macrophage migration inhibitory factor (MIF)], pulmonary edema, inflammation in the bronchoalveolar lavage fluid, and myeloperoxidase (MPO) activity and increased the activity of SOD [137]. It was also seen that in sepsis-induced ALI in rat models, curcumin markedly decreased transforming growth factor beta1 (TGF-β1) and SMAD family member 3 (SMAD3) expression, posing curcumin as protective against sepsis-induced ALI [138]. Furthermore, in sepsis-induced chronic lung injury in male albino mice models subjected to CLP, it was found that curcumin decreased inflammatory expression of IL-8, TNF-α, MIF in the bronchoalveolar fluid, and pulmonary edema and, as a whole, attenuated chronic lung injury [139].

Due to its poor bioavailability and water solubility, many formulations have been constructed to enhance the clinical effects of curcumin [275]. One formulation of a curcumin and arginine–glycine–aspartic acid nanotherapeutic was shown to target and inhibit macrophages via a decreased production of caspase-1, caspase-3, NOD-like receptor 3 (NLRP3), IL-1β, and Gasdermin D (GSDMD), which are responsible for mediating the pyroptosis seen in cytokine release syndrome and specific organ injury according to in vitro sepsis [140]. Another formulation, specifically a nanoformulation of curcumin and a cerium oxide combination with octenylsuccinic anhydride, demonstrated a reduction in inflammation and bacterial damage in in vivo sepsis models [141]. As for clinical trials of curcumin treatment for sepsis, one trial demonstrated that when a formulation of nano-curcumin was administered to septic patients in an intensive treatment unit, a marked decrease in inflammatory markers, like the erythrocyte sedimentation rate, IL-8, presepsin, and neutrophils, was observed [142]. In a randomized, double-blind, and placebo-controlled clinical trial, it was also shown that curcumin nanomicelles significantly decreased the levels of NLRP3, IFN-γ, NF-κB, IL-22, IL-17, and HMGB1 and increased forkhead box P3 (*FOXP3*) mRNA levels, thus posing as a possible supplementation for critically ill septic patients [143]. Altogether, curcumin has demonstrated a variety of effects in fighting against sepsis, ranging from treating specific organ damage to formulations that have demonstrated enhanced anti-inflammatory effects.

Curcumin has been shown to exhibit a multitude of properties in fighting COVID-19, and a plethora of clinical studies have also shown curcumin to be effective in treating COVID-19 patients [297]. Molecular docking studies have demonstrated that curcumin highly interacts with the S1 subunit of the spike glycoprotein and the ACE2 receptor of SARS-CoV-2, therefore showing promise as a prophylactic or treatment of COVID-19 [201]. Further in silico studies have also shown that curcumin can interact with the anchor site of SARS-CoV-2 Mpro, and it can thus be a potential therapeutic against COVID-19 [202]. In addition, curcumin has been shown to have binding affinity with nucleocapsid and non-structural protein (nsp) 10 of the SARS-CoV-2 proteins, thus posing as a possible combinatorial agent with drugs against COVID-19 [203]. For instance, a meta-analysis of randomized controlled trials of curcumin treatments for COVID-19 demonstrated a decrease in the overall mortality risk and showed increased benefit in fighting COVID-19 early on, within 5 days of symptom onset [204].

A nanomicellar formulation of curcuminoids, known as SinaCurcumin, was shown in various clinical studies to have improved solubility and increased oral bioavailability, thereby enhancing the therapeutic effects of curcumin [298]. One clinical study showed that SinaCurcumin decreased the levels of IL-1β and IL-6 in COVID-19 patients [205]. SinaCurcumin supplementation also demonstrated an increased activity of Tregs, which are markedly decreased in COVID-19 patients, allowing rampant inflammation, and an overall reduction in mortality [206]. Furthermore, SinaCurcumin was also shown to be effective in reducing chills, cough, olfactory, and gustatory disturbances in mild-to-moderate COVID-19 patients [207]. A non-randomized open-label trial also showed that nano-curcumin significantly resolved symptoms of fever, chills, tachypnea, myalgia, and cough and increased oxygen saturation levels in patients with mild-to-moderate COVID-19 [116]. Moreover, a combination of SinaCurcumin and catechin increased TCD4, TCD8, and Tregs and decreased Th17 cells, thus showing an improvement in inflammatory conditions in patients with COVID-19 [208]. In addition, a triple-blind, placebo-controlled, and randomized trial of COVID-19 patients treated with nanomicelles containing curcumin demonstrated a decrease in Th1 and Th17 activity and an increase in Tregs activity, thus posing a potential therapeutic to accelerate recovery in acute inflammatory COVID-19 [209]. Another clinical study showed that co-supplementation of curcumin and piperine decreased the levels of CRP and aspartate aminotransferase (AST) and increased hemoglobin levels in COVID-19 intensive care unit patients, showing promise as a supplementary therapy [210]. Furthermore, curcumin has shown potential in boosting vaccine response for COVID-19 [211]. Specifically, a study found a statistically significant increase in antibody formation in patients who received their first dose of COVID-19 vaccination along with a curcumin supplementation [211]. Moreover, in another clinical trial of curcumin in patients who had recovered from COVID-19 and were subsequently vaccinated, the phytochemical demonstrated significant anti-inflammatory activity [212]. As a whole, curcumin has been demonstrated to be a powerful agent in treating COVID-19, from its binding activity with ACE2 to its ability to ameliorate SARS-CoV-2 symptoms, thus posing as a highly promising agent that should be further explored in future regimens.

### 5.3. Luteolin

Luteolin is a flavonoid found in herbs, flowers, vegetables, and fruits with an extensive list of beneficial properties [299]. The pathway of luteolin in the body starts with the absorption of the predominant luteolin O-glycoside, which is then metabolized into glucuronidated or sulfated forms before entering the systemic circulation [300,301]. However, due to various factors, luteolin, like other flavonoids, has poor bioavailability, thus limiting its beneficial potential when orally administered [302]. Fortunately, formulations such as microemulsion systems, mono-acyl derivatives, and a combination of sodium dodecyl sulfate with luteolin nanocrystals have demonstrated enhanced bioavailability, providing promise in extracting the benefits of luteolin [303,304,305]. The general properties of luteolin include anti-cancer, anti-inflammatory, antibacterial, antiviral, and antioxidant activities [306,307,308,309,310]. In addition, studies have shown the ability of luteolin to alter chemotherapeutic effects, such as those of oxaliplatin, and help eliminate cancer cells when used in conjunction [311]. Furthermore, molecular docking studies have demonstrated that luteolin exhibits neuroprotective effects following stroke through various pathways, including the TNF pathway, and neuroprotective effects against neurodegenerative diseases, like Alzheimer’s and Parkinson’s disease [312,313]. Likewise, luteolin was shown to be effective in treating cognitive deficits in Alzheimer’s disease mouse models [314]. Luteolin has also shown immunopharmacological properties in treating specific disease states [315,316,317,318,319]. The phytochemical has also demonstrated a decrease in inflammation and colonic damage and the regulation of the gut microbiota in dextran sulfate sodium-induced ulcerative colitis rats [320]. Furthermore, luteolin, along with baicalein, has been shown to protect rat cardiomyocytes from ferroptosis induced by ischemia/reperfusion injury [321]. In addition, luteolin has shown promise in ameliorating neutrophilic asthma via inhibition of MAPK-mediated secretion of IL-36γ [322]. Overall, luteolin has demonstrated significant properties and abilities in treating clinical diseases, making it a potentially viable therapeutic regimen for further investigation.

Multiple studies have revealed that luteolin shows promising effects in fighting sepsis through a wide variety of pathways [323]. For example, luteolin has shown promise as a pre-treatment, where it restored vascular dysfunction in CLP model of septic mice through an improved expression of endothelial nitric oxide synthase (eNOS) and inducible nitric oxide synthase (iNOS) and the production of eNOS-derived nitric oxide (NO) and iNOS-derived nitrite [144]. In addition, luteolin has also exhibited improved vasoconstriction dysfunction, which is related to septic shock, in CLP mice models through the AMPK/NF-κB pathway [145]. Furthermore, in vivo models have illustrated that luteolin pre-treatment can inhibit LPS-induced lethal toxicity through the suppression of pro-inflammatory molecules like TNF-α and intercellular adhesion molecule 1 (ICAM-1), and it can decrease leukocyte tissue infiltration [146]. According to studies conducted in vivo and in vitro, luteolin can treat sepsis through the destabilization of the heat-shock protein 90 (Hsp90), which destabilizes c-Jun and Akt, thus decreasing the release of HMGB1 and its activation of the inflammatory cascade [147]. Furthermore, luteolin has been shown to increase survival rate, prevent LPS-induced organ damage, and incur improved recovery in endotoxemic mice via the inhibition of the canonical and noncanonical inflammatory pathways responsible for inducing sepsis [148].

Luteolin has demonstrated promise in treating systemic inflammation induced by sepsis and in treating organ-specific damage incited by sepsis. For example, in sepsis-induced cardiomyopathy mice models, luteolin ameliorated cardiac injury through increased autophagy via AMPK activation [149]. Luteolin has also shown the ability to inhibit LPS-induced cold-inducible RNA-binding protein (CIRP) in macrophages, thus playing a critical role in suppressing sepsis-induced lung injury in neonatal mice [150]. Furthermore, septic mice models demonstrated that luteolin pre-treatment ameliorated sepsis-induced ALI through the inhibition of oxidative stress, NF-κB, ICAM-1, and iNOS [151]. Moreover, pre-treatment with luteolin in CLP-induced sepsis mice models also showed increased Treg frequency and, thus, IL-10 production and diminished caspase-11-dependent pyroptosis in alleviating sepsis-induced lung injury [318]. In addition, LPS-induced septic mice models have revealed that luteolin can reverse LPS-induced hepatic injury, possibly through the decreased production of HMGB1 and its role in the purinergic receptor P2X 7 (P2X7R)-receptor for the advanced glycation end products (RAGE)-TLR4 axis, which plays a part in hepatic injury [152]. Luteolin pre-treatment has also shown protective effects against AKI induced by LPS in septic mice [153]. The traditional Chinese medicine Lianhua Qingwen contains luteolin as one of its four active components and is effective in treating sepsis-induced ALI through the inhibition of the p53-mediated apoptotic pathway [154]. Altogether, luteolin has exhibited an extensive array of properties in treating sepsis by modulating systemic inflammatory effects and/or alleviating organ-specific damage, thus acting as a viable therapeutic regimen in ameliorating the disease condition and improving patient outcomes.

Luteolin has exhibited an extensive list of properties in fighting COVID-19 and its various specificities. For instance, molecular docking studies have shown that luteolin binds to the 3CLpro, papain-like protease (PLpro) proteases, and spike protein of SARS-CoV-2 [213]. In addition, in vitro studies have also illustrated luteolin’s ability to bind to RdRp of SARS-CoV-2 [214]. Molecular docking studies have further shown that luteolin holds antiviral potential against SARS-CoV-2 by revealing its ability to bind to 3CL protease and MAPK1 [215]. More molecular docking studies have shown that luteolin exhibits binding affinity for ACE2 receptor and transmembrane protease serine 2 (TMPRSS2), along with Mpro and RdRp, in COVID-19 [216]. Furthermore, molecular docking studies also revealed that luteolin, along with abyssinone II, portrayed the highest binding affinity for the Mpro/3CLpro, PLpro, and ACE2 targets of COVID-19 in comparison to other flavonoids, drugs, and molecules [217]. Not only does luteolin demonstrate activity against SARS-CoV-2 but also its comorbidities. For instance, a system pharmacology and bioinformatics analysis revealed that luteolin holds properties in terms of fighting against COVID-19/asthma comorbidity through its ability to regulate oxidative stress, inflammation, viral defense, immune response, and the cell cycle [218].

As for clinical trials, an extensive list of studies have examined luteolin’s ability to fight chronic symptoms following COVID-19 infection. One double-blind and placebo-controlled trial revealed that patients suffering from chronic olfactory dysfunction after COVID-19 experienced greater olfactory recovery when they received olfactory training, co-ultramicronized palmitoylethanolamide, and luteolin together compared to their individual therapy [219]. In further support, one randomized–controlled pilot study found that COVID-19 patients who reported persistent olfactory impairment even after 90 days from SARS-CoV-2 negative testing had improved olfactory function after receiving weekly olfactory rehabilitation and palmitoylethanolamide and luteolin, including the ability to identify and discriminate smells [220]. In addition, co-ultramicronized palmitoylethanolamide and luteolin have also been shown to increase long-interval intracortical inhibition (LICI) and long-term potentiation (LTP)-like cortical plasticity in patients suffering from persistent cognitive dysfunction and fatigue post COVID-19, thus showing promise as a therapeutic for cognitive dysfunction in long COVID-19 syndrome [221]. Furthermore, a longitudinal study found that in patients with olfactory dysfunction and mental clouding/brain fog from long COVID-19, ultra-micronized palmitoylethanolamide and luteolin and olfactory training improved both chronic symptoms [222]. Altogether, numerous studies have revealed luteolin’s array of abilities in fighting COVID-19, from formulations with palmitoylethanolamide to treating COVID-19 comorbidities, thus showing promise in the therapeutic approach for COVID-19.

### 5.4. Apigenin

Apigenin is a flavonoid abundantly found in fruits, vegetables, nuts, and herbs with a high content, especially in chamomile, celery, vine spinach, artichokes, and oregano [324]. Because of its lipophilic nature, it has greater permeability through the plasma membrane [325,326]. However, its poor water solubility and deactivation in the acidic environment of the gastrointestinal tract limits its bioavailability [325,327,328]. The conjugate of apigenin with β-glycosides has increased bioavailability and is absorbed through the intestinal route via interactions with gut microbiota [329]. In addition, various delivery systems such as emulsions, nanostructured lipid carriers, hydrogels, and liposomes have been constructed for better stability and enhanced digestion and absorption of apigenin [327,330,331]. Apigenin possesses a wide range of biological and therapeutic properties including antioxidant, anti-inflammatory, anti-cancer, and antimicrobial effects [332,333,334,335,336]. Apigenin has been demonstrated to alleviate nephropathy, pancreatic beta cell dysfunction, genitourinary dysfunction, cardiomyopathy, and liver dysfunction in diabetes [337,338,339,340,341]. It has been shown to exhibit protective actions against cardiometabolic diseases including obesity, diabetes, hypertension, and cardiovascular diseases [342,343,344,345,346,347]. Furthermore, the neuroprotective effects of apigenin on cerebrovascular diseases have been well established through various studies. [348,349,350,351]. Human clinical studies have also been performed using apigenin-containing dietary supplements against various ailments. [352,353,354,355]. These encouraging findings highlight the implications of apigenin for future therapeutic interventions.

Through the various therapeutic properties discussed in the previous section, apigenin has been reported to have protective roles in sepsis treatment and organ protection [356]. In a rat model of polymicrobial sepsis, apigenin inhibited oxidative stress and inflammatory cell damage through the modulation of inflammatory cytokines and antioxidant enzyme activities, thereby improving sepsis-induced lung injury [155]. Similarly, in another study, apigenin was able to decrease the levels of pro-inflammatory cytokines, TNF-α, IL-1-β, IL-6, and TGF-β and increase the level of an anti-inflammatory cytokine, IL-10, in a rat model of CLP [156]. In addition, the spleen tissue of these rats supplemented with apigenin showed inhibition of the NF-κB pathway, which rescued the spleen from sepsis-induced oxidative injury. Another study, which evaluated the effects of apigenin supplementation on heart injury in an LPS-induced endotoxemic rat model, showed that apigenin exhibited prominent cardioprotective properties by inhibiting the myocardial apoptosis and inflammatory signaling through the sphingosine kinase 1/sphingosine-1-phosphate (SphK1/S1P) signaling pathway. [157]. The study showed that apigenin treatment could decrease the levels of creatine kinase-MB (CK-MB), LDH, TNF-α, IL-6, and IL-1β in serum along with an inhibition of proapoptotic caspases 3 and 9 and the activation of antiapoptotic Bcl-2. In another model of endotoxin-induced myocardial injury, apigenin was able to alleviate the LPS-induced cardiac injury by modulating redox homeostasis, inflammatory signaling through NF-κB, and autophagy via transcription factor EB (TFEB), vacuolar protein sorting-associated protein 11 (Vps11), and microtubule-associated proteins 1A/1B light chain 3B (Map1lc3) [158]. Apigenin has also been reported to alleviate LPS-induced ALI through its antioxidant and anti-inflammatory properties that modulate the molecular signaling of NF-κB, nuclear factor erythroid 2-related factor 2 (Nrf-2), and peroxisome proliferator-activated receptor gamma (PPARγ) [159,160,161]. In addition, in an LPS/d-galactosamine (d-GalN)-induced acute liver failure model in mice, apigenin showed remarkable hepatoprotective effects by improving antioxidant enzyme activities and decreasing hepatotoxicity markers and pro-inflammatory cytokines [162]. The study demonstrated the modulation of the NF-κB and apoptotic signaling pathways in apigenin-supplemented mice. Studies have shown that apigenin has a modulatory effect on the TLR-4, MAPK, and NF-κB pathways in LPS–induced ALI [163,164]. In human lung epithelial cells, apigenin was able to suppress LPS-induced pro-inflammatory cytokines and AP-1 factors, displaying its importance in the treatment of lung inflammatory diseases [165]. In addition, apigenin was able to protect mice treated with a lethal dose of LPS by modulating the apoptosis, infiltration of inflammatory cells, and accumulation of chemotactic factors in the liver [166]. Apigenin supplementation significantly decreased LPS-induced apoptosis, ROS production, and caspase 3 activity in endothelial cells along with a normalization of mitochondrial function [167].

Various experimental approaches have demonstrated the therapeutic utility of apigenin against COVID-19 infection. Molecular docking studies have identified apigenin 7-glucoside-4′-p-coumarate as the best candidate for SARS-CoV-2 Mpro inhibition [223]. An integrated in silico pharmaco-bioinformatics approach elucidated the role of apigenin in the management of the synergetic incidence of COVID-19 and human immunodeficiency virus (HIV) [224]. The study identified apigenin as an effective therapeutic agent with minimal adverse effects against both of these two viral infections. A proteolytic assay followed by an induced-fit docking experiment showed that another derivative of apigenin, apigenin-7-O-rhamnoglucoside, has prominent inhibitory activity in the catalytic domain of SARS-CoV 3CLpro [225]. Apigenin has been presented as the main constituent of *Moringa oliefera* with the highest binding affinities against nsp9 and nsp10, which mediate the neutrophils chemotaxis and inflammatory response in COVID-19 [226]. Also, apigenin has been identified as an active component of *Ginkgo biloba* and *Rosmarinus ofcinalis* L., which have an inhibitory effect on SARS-CoV-2 3CLpro and Mpro, respectively [227,357]. Also, apigenin-7-O-rutinoside achieved the highest binding affinities towards nsp16/10 complex when docked against the main targets involved in SARS-CoV-2 infection [229]. Structure–activity studies based on molecular docking and cell-based replication assays have demonstrated the effect of apigenin on the protease inhibition and viral replication of SARS-CoV-2 [230]. In one of these studies, the infection of African green monkey kidney Vero E6 cells with SARS-CoV-2 USA-WA1/2020 isolate in the presence of apigenin showed an inhibition of the virus infection. In another study, SARS-CoV-2 B.1 isolated from Vero E6 cells in nasopharyngeal swabs of a COVID-19 patient were incubated with human lung epithelial cells (Calu-3) in the presence of apigenin. The treatment with apigenin effectively reduced the virus infection and significantly decreased levels of the inflammatory cytokine TNF-α in the cell culture supernatant [231]. Hence, the aforementioned studies suggest that in addition to the remarkable ability to alleviate the complications of sepsis-associated organ damage, apigenin can act as a potential phytochemical that can inhibit viral infection in COVID-19. This will guide the development of future therapeutic interventions using apigenin against sepsis associated with COVID-19.

### 5.5. Resveratrol

Resveratrol is a polyphenolic compound produced by plants in response to external stressors such as ultraviolet radiation, mechanical damage, or fungal infection [358]. It is ubiquitously present in fruits such as grapes, raspberries, blueberries, plums, and peanuts, as well as roots, stems, and leaves, with the highest concentrations found in the Japanese knotweed *Polygonum japonicum*, which is used in tea products and is renowned for its potent antioxidant activity [168,169]. The chemical structure of resveratrol confers low water solubility, which affects its absorption [359,360]. In the intestine, this phytochemical is absorbed either by passive diffusion or through interactions with membrane transporters like integrins. Once in the bloodstream, resveratrol undergoes hepatic metabolism, producing conjugated sulfates and glucuronides that retain their biological activity [359,361,362]. Resveratrol has been shown to exhibit a wide range of clinically relevant properties, including anti-inflammatory, antiviral, antibacterial, antifungal, and anti-tumor activities [359,363,364,365,366,367,368]. Polyphenols, including resveratrol, enhance the expression of silent information regulator SIRT1, inhibit NF-κB activation, and downregulate nitric oxide synthase (NOS), adhesion molecules, and TNF-α [170,171]. Notably, it has shown effectiveness in mitigating sepsis-related and COVID-19-associated organ damage [369,370]. These characteristics suggest that resveratrol is a potential candidate for COVID-19-associated sepsis treatment and offers a guideline for future research and clinical applications.

It has been shown that resveratrol has a protective effect on septic shock [371]. In endotoxemia models, it reduced oxidative damage by modulating erythrocyte lipid peroxidation and catalase activity, inhibiting NO release, downregulating malondialdehyde levels, and maintaining iron homeostasis [172]. Additionally, resveratrol activates AMPK in LPS-stimulated macrophages via the calcium-/calmodulin-dependent protein kinase kinase (CaMKK) pathway, enhancing phagocytosis, regulating inflammation, and preventing endotoxin tolerance by inhibiting the expression of anti-inflammatory IL-1 receptor-associated kinase-M (IRAK-M) and inositol 5′ polyphosphatase 1 (SHIP-1) induced by LPS [173]. In models of LPS-induced sepsis, resveratrol has been shown to alleviate ALI by suppressing inflammation and apoptosis of alveolar macrophages, inhibiting the production of TNF-α, IL-6, and IL-1β, which are associated with the inhibition of NF-κB, p38, and extracellular signal-regulated kinase (ERK) signaling pathways [174]. It also protects against CLP-induced ALI/ARDS by regulating phospholipid scramblase 3 (PLSCR-3)-mediated mitochondrial dysfunction and mitophagy by modulating autophagy-related (ATG) and microtubule-associated protein 1A/1B-light chain 3 (LC3-I/II) and P62 [175].

Resveratrol has not only demonstrated a potential effect for sepsis-induced ALI/ARDS but also sepsis-induced cardiomyopathy (SIC), a severe myocardial dysfunction secondary to septicemia [176,372]. Resveratrol’s cardioprotective effects in SIC are mediated through the inhibition of ferroptosis via the upregulation of the SIRT1/Nrf2 signaling pathways, leading to improved cardiac function and reduced myocardial damage, impaired mitochondria, and lipid peroxidation [176]. It also benefits vascular dysfunction by upregulating eNOS expression through Ras-related C3 botulinum toxin substrate 1 (Rac-1) and HIF-1α inhibition [177]. In septic models, resveratrol has been shown to inhibit reactive nitrogen species in kidney tissue, restoring renal microcirculation and protecting the tubular epithelium [178]. Also, it increased survival rates in septic rats by mitigating AKI through inflammatory factor inhibition and promoted NF-κB-p65 de-acetylation by upregulating SIRT1 and deactivating the long noncoding RNA metastasis-associated lung adenocarcinoma (MALAT1)/miR-205 axis [179,180]. The neurological system is also protected by resveratrol, as evidenced by a study that demonstrated that resveratrol suppressed the LPS-induced degradation of the inhibitor of nuclear factor kappa B alpha (IkappaBα), iNOS expression, and p38 MAPK phosphorylation in microglial cells, suggesting its potential in treating neurodegenerative diseases associated with microglial activation [170].

Given its antiviral, anti-inflammatory, and antioxidant properties, resveratrol has been demonstrated as an advantageous antiviral therapy for SARS-CoV-2 infection [118,232]. In a study conducted in air–liquid-interface-cultured human primary bronchial epithelial cells, resveratrol and its metabolically more stable structural analog, pterostilbene, showed potential antiviral activity, inhibiting virus replication [232]. A similar study revealed that resveratrol inhibited the replication of SARS-CoV-2 in cultured Vero cells [118]. Resveratrol also inhibited viral replication in cultured fibroblasts isolated from lung tissue (MRC-5) [233]. Additionally, it has been shown that resveratrol has low cytotoxicity and acts as a specific inhibitor of SARS-CoV-2’s 3-chymotrypsin-like protease and PLpro [234]. Gene analysis suggests that resveratrol targets the *IL-17, NF-κB*, and *TNF* signaling pathways in COVID-19 therapy [235]. Clinical trials have reported that patients with resveratrol supplementation had a lower incidence of hospitalization, COVID-related emergency room visits, and pneumonia compared to a placebo supplementation in outpatients with mild COVID-19 [236]. Hence, the pharmacological profile of resveratrol highlights its potential as a therapeutic agent in managing COVID-19-associated sepsis. The broad spectrum of resveratrol’s protective effects and its favorable safety profile and natural origin highlight its importance as a potential therapeutic agent. This underscores the need for rigorous clinical research to validate its efficacy and optimize its clinical application, paving the way for resveratrol to be integrated into treatment protocols for COVID-19-associated sepsis.

### 5.6. Naringenin

Naringenin is considered one of the most important flavonoids, mainly as a flavanone, due to its potential biological activities such as antioxidant, anti-inflammatory, and antiviral properties [373]. This phytochemical is extensively found in various fruits and vegetables, either in a free form or as glycosides or acyl glycosides; the highest concentrations are reported in grapefruit, lemon, oranges, bergamot, and tomatoes, contributing to its substantial dietary intake [181]. Naringenin absorption occurs via passive diffusion and active transport in the gastrointestinal tract [374,375,376]. Post absorption, it binds to albumin and is distributed to highly perfused organs [377,378]. Due to its numerous health benefits, naringenin is incorporated into various pharmaceutical formulations to enhance human health [379]. Studies have shown that naringenin can improve sepsis-related and COVID-19-associated organ damage [182,237,380,381]. These properties suggest that naringenin is a promising candidate for treating COVID-19-associated sepsis, offering a guideline for future research and clinical applications.

Naringenin exhibits protective roles in sepsis treatment and organ protection [380]. It inhibits the leukotriene B4 (LTB4)/leukotriene B4 receptor 1 (BLT1) receptor, attenuating inflammation and apoptosis by activating the AMPK signaling pathway and inhibiting NF-κB signaling and mitochondrial damage in septic cardiac dysfunction models [182]. In LPS-induced injury in normal human bronchial epithelium models, naringenin reduced inflammation by decreasing the secretion of TNF-α, IL-6, SOD, NOS, MPO, and NO while also attenuating MAPK activation by downregulating the phosphorylation of ERK1/2, JNK, and p38 MAPK [183]. Another study revealed that naringenin protects against sepsis-related lung damage through AMPK-activating transcription factor 3 (ATF3)-dependent negative regulation of the LPS/TLR4 signaling pathway, suppressing the expression of TNF-α, IL-6, TLR4, iNOS, cyclo-oxygenase-2 (COX2), and NOX2 [184]. In addition, in an LPS-induced ALI in a mouse model, naringenin increased survival rates, alleviated pulmonary edema, and reduced lung vascular leakage through its antioxidative and anti-inflammatory activities, inhibiting the PI3K/AKT pathway [185]. Studies have shown that naringin, the glycoside form of naringenin, reduces the production of inflammatory cytokines in sepsis models via various signaling pathways, such as Kelch-like ECH-associated protein 1 (KEAP1)/Nrf-2/heme oxygenase-1 (HO-1) [188], Ras homolog family member A-associated protein kinase/NF-κB/Myosin light-chain kinase (MLCK)/myosin light chain (MLC) (RhoA/ROCK/NF-κB/MLCK/MLC [187], PI3K/AKT [188], and MAPK/AMPK [382]. Naringin also exhibits anti-inflammatory, anti-apoptotic, and antioxidant effects against sepsis injury by decreasing M1 phase polarization and increasing M2 phase polarization via the PPARγ/miR-21 axis, underscoring its potential benefits in septic conditions [189].

Naringenin has been included in therapeutic studies for COVID-19, and through pharmacokinetic studies, it has demonstrated efficacy in restricting viral attachment to the host cells via the inhibition of the spike glycoprotein or its cellular receptor [201]. Docking-based virtual screening suggests that naringenin acts by inhibiting 3CLpro and decreasing ACE receptor activity [237]. Another molecular docking study indicates that naringenin exerts a therapeutic effect on one of the most significant genes, *AKT1*, involved in lung injury, lung fibrogenesis, and viral infections [238]. Additionally, a molecular docking study revealed that naringenin might act by targeting proteins such as nsp3, nsp7, nsp8, and nsp12 from SARS-CoV-2 and via the inhibition of RNA polymerase [239]. It also modulates human coronavirus infections by targeting the molecular target two-pore channel 2 (TPC2) in Vero E6 cells [240]. Given these promising characteristics, naringenin is a strong candidate for future clinical trials targeting COVID-19-associated sepsis. Its multi-faceted mechanism of action, combined with its favorable safety profile and natural dietary presence, supports its potential to be developed as an adjunctive therapy. Further clinical research is warranted to fully elucidate its therapeutic benefits and optimize its application in treating the complex pathophysiology of COVID-19-associated sepsis, thereby enhancing patient outcomes and providing a robust addition to current treatment strategies.

## 6. Conclusions

Even though many phytochemicals have shown promising therapeutic potential, the inadequate knowledge of their mode of action, potential adverse reactions, contraindications, and interactions with other drugs and foods pose potential challenges to their use as therapeutic agents. Proper scientific investigations are essential to determine the best phytochemical combinations and dosages required to ensure standardized and high-quality phytochemical formulations. In addition, as discussed previously, numerous phytochemicals with potential pharmacological actions have limited bioavailability owing to their poor solubility and stability features, which, in turn, hinder their therapeutic potential. The magnitude of the bioavailability can be correlated with the pharmacokinetics of the phytochemicals, including digestion, epithelial absorption, distribution, biochemical degradation, and excretion [383,384]. Understanding the problems associated with the bioavailability of specific phytochemicals may result in strategies to overcome their constraints. Advanced approaches in novel delivery systems have been developed to modulate the pharmacokinetics of phytochemicals in order to enhance their bioavailability, including their co-administration with other compounds [273], drug-loaded nanosized drug carriers, such as polymeric nanoparticles [385], liposomes [386], dendrimers [387], micelles [388], and implants of drug-loaded polymers [389]. Therefore, strategies for optimizing the protective efficacy of dietary phytochemicals warrant detailed scientific validation. The present review highlights the imperative need to promote the utility of phytochemicals as functional foods and nutraceuticals in COVID-19-associated sepsis by emphasizing the pharmacological properties of some potent phytochemicals commonly present in our daily diet.

**Figure 2:**
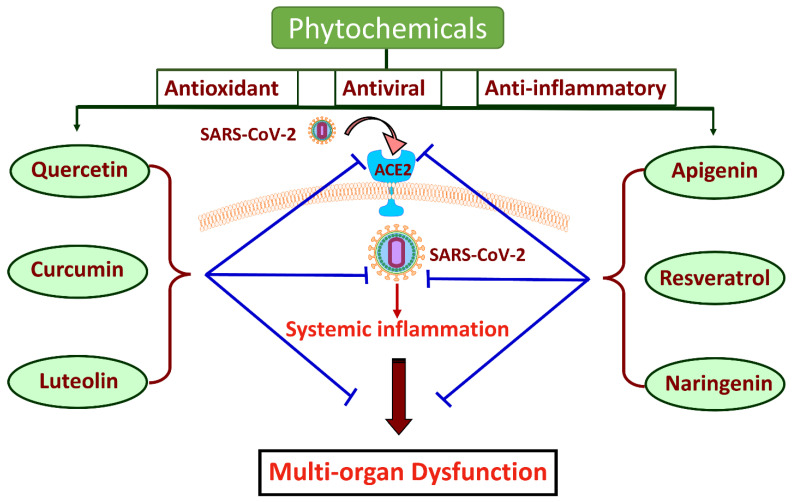
Schematic representation showing the protective effects of phytochemicals in sepsis associated with COVID-19. Phytochemicals effectively inhibit SARS-CoV-2 viral infection and attenuate systemic inflammation that affects multiple organ functions in sepsis through their remarkable pharmacological properties. Abbreviations: severe acute respiratory syndrome coronavirus 2 (SARS-CoV-2), angiotensin-converting enzyme 2 (ACE2).

In summary, the present review outlines the pathogenic progression of sepsis in COVID-19 and accentuates the importance of phytochemicals for its possible mitigation (Figure 2). As we discussed, the clinical manifestations of COVID-19 overlap with the pathophysiology of sepsis, potentially resulting in lethal multi-organ system damage. COVID-19-associated inflammatory progression in combination with impaired host response to infection and excessive oxidative stress further exacerbates the septic damage. The heterogeneity of sepsis pathogenesis among COVID-19 patients and the risks of secondary infections and other adverse effects of current treatment strategies imply the necessity of therapeutic approaches using medicinal plants and their phytoconstituents. Phytochemicals such as functional foods and nutraceuticals promote health and have incredible therapeutic potential to cure chronic diseases. We believe that the studies consolidated in this review illustrate the remarkable properties of some potent phytochemicals in alleviating the multi-organ complications of sepsis and their pivotal role in fighting against COVID-19 infection. Thus, this review emphasizes the need for advanced research to validate the effectiveness of these phytochemicals in order to integrate them into treatment protocols for COVID-19-associated sepsis.

## Figures and Tables

**Table 1:** Effect of phytochemicals on the pathophysiological complications associated with sepsis.

Phytochemical	Sources	Experimental Models	Physiological Effects	References
Quercetin	Citrus fruits, apples, onions, broccoli, parsley, tea, red wine, olive oil, grapes, dark cherries, and dark berries.	In vitro: LPS-treated cells (RAW264.7, human alveolar epithelial A549 cells, murine lung epithelial cells, macrophages, and H9C2 cells). In vivo: CLP in rats and mice; LPS-treated mice; E. coli K1 treatment in mice. In silico: Network pharmacology.	⇩ Inflammatory signaling ⇩ NF-κB, TXNIP, HIF-1, TNF, NOD-like receptor, NOX2, HMGB1, and CXCL8 ⇩ ROS and oxidative damage ⇩ ER stress ⇩ Cardiomyocyte pyroptosis and myocardial injury ⇩ Pro-inflammatory cytokines ⇩ Mortality ⇩ Lung edema, congestion, and hemorrhage ⇩ Apoptosis ⇩ Bacteria abundance and endotoxin levels ⇧ Mitochondrial function ⇧ SIRT1, AMPK ⇧ Anti-inflammatory cytokine levels ⇧ Antioxidant enzymes (SOD, CAT, and APX)	[119,120,121,122,123,124,125,126,127,128,129]
Curcumin	Turmeric	In vitro: LPS-treated cells (HL-1 cells, RAW264.7) In vivo: LPS-treated mice and rats; CLP in mice. In silico: Gene expression matrix. Clinical trial: Human patients.	⇩ Inflammatory signaling ⇩ Oxidative stress ⇩ STAT3, NF-κB, p38, TNF-α, IL-1β, IL-6, IL-8, MIF, MPO, MDA, IFN-γ, TLR1, caspase-1, caspase-3, NLRP3, IL-1β and GSDMD, mTORC1, TGF-β1, SMAD3, Raptor, CD39+ Tregs, Cathepsin B, IL-22, IL-17, and HMGB1. ⇩ Presepsin ⇩ AST, ALT, BUN, and creatinine ⇩ Muscle protein breakdown, pulmonary edema, injury, and apoptosis of the liver and kidneys ⇩ Mortality ⇧ SOD, IL-10, miR-183-5p, p-PI3K, p-AKT, SIRT1, PGC1α, Tfam, Nrf2, and *FOXP3* ⇧ Mitochondrial function, cardiac function, and survival	[130,131,132,133,134,135,136,137,138,139,140,141,142,143]
Luteolin	Celery, parsley, broccoli, onion leaves, carrots, peppers, cabbages, apple skins, and chrysanthemum flowers.	In vitro: LPS-treated cells (RAW264.7 macrophages, peritoneal macrophages, HepG2 cells). In vivo: CLP in mice; LPS-treated mice; CSI in mouse pups. In silico: Network pharmacology.	⇩ Inflammatory signaling ⇩ Oxidative stress ⇩ Pro-inflammatory cytokines ⇩ NF-κB, p-p65/p65, p-IκBα/IκBα, ICAM-1, HMGB1, c-Jun, Hsp90, TLR-4, caspase-1/4/5/11, Bax, CIRP, HIF-α, NLRP3, GSDMD, IL-1α, p53, IL-6 and IL-1β, TNF-α, MCP-1, iNOS, and nitrite ⇩ ALT, AST, BUN, and serum creatinine ⇩ Leukocyte infiltration in the liver and lungs ⇩ Blood and lung tissue bacterial counts ⇩ Lung edema, AKI, mitochondrial dysfunction, lung and cardiac apoptosis ⇧ eNOS and nitric oxide ⇧ Survival rate, cardiac function, autophagy, and lung architecture ⇧ SAP, DAP, MAP, and vasoconstriction function ⇧ ADRA1A, p-AMPK/AMPK, Treg, IL-10, and Bcl-2 ⇧ Antioxidant enzyme activities (SOD, CAT)	[144,145,146,147,148,149,150,151,152,153,154]
Apigenin	Chamomile, parsley, celery, onions, oranges, thyme, oregano, and basil.	In vitro: LPS-treated cells (H9c2 cells, RAW264.7 macrophages, human lung A549 cells, bovine aortic endothelial cells). In vivo: LPS-treated mice and rats.	⇩ Inflammatory signaling ⇩ ROS and oxidative stress ⇩ TNF-α, TGF-β, IL-1β, IL-6, IL-2, CD3, CD68, NF-κB, caspase-3, cleaved caspase-9, Bax, MIP-1α, MPO, MDA, PGE2 and MIP-2, iNOS, COX-2, p38, ERK1/2, JNK, c-Jun, c-Fos, and JunB, TLR4, and TRPC6 ⇩ NO, nitrotyrosine, and protein carbonyls ⇩ ALT, AST, ALP, γ-GT, CRP, total and direct bilirubin levels ⇩ MAPK pathway, SphK1/S1P signaling pathway proteins, and apoptosis in the heart ⇩ Cardiac injury (CK-MB, LDH, DNA fragmentation, cTnI, cMLC1, and PARP activity) ⇩ Apoptosis, inflammatory cell infiltration, and chemotactic factor accumulation in the lungs ⇩ Mitochondrial dysfunction ⇧ SOD, CAT, GSH, T-AOC, HO-1, IL-10, Bcl-2, Nrf-2, and PPARγ ⇧ Autophagy	[155,156,157,158,159,160,161,162,163,164,165,166,167]
Resveratrol	Grapes, raspberries, blueberries, plums, peanuts, roots, stems, leaves, and teas.	In vitro: LPS-treated cells (rat cortical microglia, mouse microglial cell line N9, human umbilical endothelial cells, macrophages, MH-S cells). In vivo: LPS-treated rats; CLP in mice.	⇩ Inflammatory signaling ⇩ Pro-inflammatory cytokines ⇩ NF-κB, nitric oxide synthase ⇩ p38 MAPK/NF-κB pathway ⇩ Lipid peroxidation, NOS ⇩ IRAK-M, SH2, SHIP1, PLSCR-3, ATG5, ATG7, LC3-I/II, P62, MALAT-1 ⇩ Apoptosis ⇩ Interstitial edema ⇩ ALI ⇩ AKI ⇩ Myocardial damage, cardiac dysfunction, cardiomyopathy ⇩ Mortality ⇧ SIRT1/Nrf2, eNOS ⇧ CAT ⇧ AMPK/CaMKK ⇧ Mitochondrial function and renal microcirculation	[168,169,170,171,172,173,174,175,176,177,178,179,180]
Naringenin	Grapes, lemons, oranges, bergamot, and tomatoes.	In vitro: Human bronchial epithelium; LPS-stimulated murine macrophages; MODE-K cells. In vivo: LPS-treated mice and rats.	⇩ Inflammatory signaling ⇩ Pro-inflammatory cytokines ⇩ NF-κB pathway ⇩ AMPK/PGC1α ⇩ SOD, NOS, MPO, NO ⇩ ERK1/2/JNK/p38 MAPK, TLR4, iNOS, COX2, NOX2, PI3K/AKT, Keap1 ⇩ ROS ⇩ Macrophage 1 phase polarization ⇩ Apoptosis ⇩ Lung vascular leak ⇩ Pulmonary edema ⇩ ALI ⇩ Intestinal injury ⇧ Nrf2, HO-1, RhoA/ROCK/NF-κB/MLCK/MLC, PPARγ ⇧ Mitochondrial function ⇧ Cardiac function Survival	[181,182,183,184,185,186,187,188,189]

Abbreviations: nuclear factor kappa B (NF-κB), thioredoxin-interacting protein (TXNIP), hypoxia-inducible factor-1 (HIF-1), tumor necrosis factor (TNF), nucleotide-binding and oligomerization domain (NOD)-like receptor, nicotinamide adenine dinucleotide phosphate oxidase 2 (NOX2), high-mobility group box 1 (HMGB1), C-X-C Motif Chemokine Ligand 8 (CXCL8), reactive oxygen species (ROS), endoplasmic reticulum (ER), Sirtuin 1 (SIRT1), AMP-activated protein kinase (AMPK), superoxide dismutase (SOD), catalase (CAT), ascorbate peroxidase (APX), signal transducer and activator of transcription 3 (STAT3), interleukin (IL), macrophage migration inhibitory factor (MIF), myeloperoxidase (MPO), malondialdehyde (MDA), Interferon-gamma (IFN-γ), toll-like receptor 1 (TLR1), NOD-like receptor 3 (NLRP3), Gasdermin D (GSDMD), mammalian target of rapamycin complex 1 (mTORC1), transforming growth factor-beta (TGF-β), SMAD family member 3 (SMAD3), cluster of differentiation (CD), T regulatory cells (Tregs), Aspartate Transferase (AST), Alanine Transaminase (ALT), blood urea nitrogen (BUN), phosphatidylinositol 3-kinase (PIK3), peroxisome proliferator-activated receptor-γ coactivator 1-α (PGC1α), mitochondrial transcription factor A (Tfam), nuclear factor-erythroid factor 2-related factor 2 (Nrf2), forkhead box P3 (FOXP3), inhibitor of nuclear factor kappa B (IκB), intercellular adhesion molecule 1 (ICAM-1), heat-shock protein 90 (Hsp90), cold-inducible RNA-binding protein (CIRP), monocyte chemoattractant protein-1 (MCP-1), inducible nitric oxide synthase (iNOS), endothelial nitric oxide synthase (eNOS), systolic arterial pressure (SAP), diastolic arterial pressure (DAP), mean arterial pressure (MAP), alpha-1A and beta-2 adrenergic receptors (ADRA1A), macrophage inflammatory protein (MIP), prostaglandin E2 (PGE2), cyclo-oxygenase-2 (COX-2), extracellular signal-regulated kinase1/2 (ERK 1/2), transient receptor potential cation channel subfamily C member 6 (TRPC6), alkaline phosphatase (ALP), gamma-glutamyltransferase (γ-GT), C-reactive protein (CRP), sphingosine kinase 1 (SphK1), sphingosine-1-phosphate (S1P), creatine kinase-MB (CK-MB), lactate dehydrogenase (LDH), cardiac troponin I (cTnI), cardiac myosin-light chains 1 (CMLC-1), poly (ADP-ribose), polymerase (PARP), glutathione (GSH), total antioxidant capacity (T-AOC), heme oxygenase-1 (HO-1), peroxisome proliferator-activated receptor gamma (PPAR-γ), calcium-/calmodulin-dependent protein kinase kinase (CaMKK), interleukin-1 receptor-associated kinase-M (IRAK-M), Src homology 2 (SH2), inositol 5′ polyphosphatase 1 (SHIP-1), phospholipid scramblase 3 (PLSCR-3), microtubule-associated protein 1A/1B-light chain 3 (LC3-Ⅰ/Ⅱ), autophagy-related genes (*ATG*), mitogen-activated protein kinase (MAPK), c-Jun NH2-terminal kinase (JNK), Kelch-like ECH-related protein 1 (Keap1), Ras homolog family member A-associated protein kinase (RhoA-ROCK), myosin light-chain kinase (MLCK), myosin light chain (MLC), metastasis-associated lung adenocarcinoma (MALAT1), lipopolysaccharides (LPSs), cecal ligation and puncture (CLP), cecal slurry injection (CSI), acute kidney injury (AKI), acute lung injury (ALI).

**Table 2:** Effect of phytochemicals on the pathophysiological complications associated with COVID-19.

Phytochemical	Experimental Models	Pharmacological Effects	References
Quercetin	In vitro: Antiviral activity; activity-based experimental screening; SARS-CoV-2-infected green monkey kidney Vero E6 cells and human colon carcinoma Caco-2 cells; human embryonic kidney HEK293 co-expressing SARS-CoV-2 spike (S) protein and ACE2; H1975-ACE2; 293T-ACE2; BEAS-2B-ACE2 cells. In vivo: SARS-CoV-2-infected hamsters and mice. In silico: Molecular docking; network pharmacology; protein–protein interaction network. Clinical trials: Human patients.	Inhibits SARS-CoV-2 3CLpro, ACE2 Blocks TLR, HIF-1alpha, VEGF, TNF, and apoptosis pathways Inhibits SARS-CoV-2 replication, formation of syncytia, production of the S2’ fragment of the spike protein, furin Eliminates virus-induced senescence cells, mitigated lung disease, and reduced inflammation Reduces virus infection Inhibits PDE4 and SARS-CoV-2 Mpro Speeds recovery and reduces the severity of symptoms Reduces serum levels of ALP, q-CRP, and LDH Increases in hemoglobin level and respiratory rate	[190,191,192,193,194,195,196,197,198,199,200]
Curcumin	In silico: Network pharmacology; molecular docking. Clinical trials: Human patients.	Interacts with SARS-CoV-2 Mpro, spike glycoprotein, nucleocapsid phosphoprotein, nsp10, RdRp, and ACE2 receptor Reduces mortality rate Decreases IL-1β, IL-6, CRP, AST Increases hemoglobin Improves immune response (antibody formation, TCD4+, TCD8+, Treg cells, FoxP3, IL-10, IL-35, and TGF-β) Speeds recovery, reduces severity of symptoms Accelerates the recovery of the acute inflammatory phase Anti-inflammatory and prophylactic properties	[116,201,202,203,204,205,206,207,208,209,210,211,212]
Luteolin	In silico: Network pharmacology; molecular docking. Clinical trials: Human patients.	Binds to SARS-CoV-2 3CLpro, PLpro proteases and spike protein, 3CL protease, MAPK1, ACE2 receptor, TMPRSS2, Mpro, RdRp Targets TP53, AKT1, ALB, IL-6, TNF, and VEGFA Regulates TLR-4, MAPK, TNF, AGE/RAGE, EGFR, HIF-1, and PI3K–AKT Regulates inflammation, virus defense, cell growth, cell replication, immune responses, oxidative stress, and blood circulation Greater olfactory recovery, memory, and ability to identify and discriminate smells. Enhances GABA-ergic transmission and reduces neuroinflammation Increases LICI- and LTP-like cortical plasticity	[213,214,215,216,217,218,219,220,221,222]
Apigenin	In vitro: SARS-CoV-2-infected African green monkey kidney Vero E6 cells; human lung epithelial Calu-3 cells. In silico: Network pharmacology; molecular docking.	Inhibits SARS-CoV-2 Mpro, 3CLpro, Interacts with MAPK3, RELA, MAPK1, EP300, and AKT1 Binds to nsp9, nsp16, and nsp10 Inhibits proteases and viral replication Decreases inflammatory cytokine (TNF-α)	[223,224,225,226,227,228,229,230,231]
Resveratrol	In vitro: Human primary bronchial epithelial cells; Vero cells; MRC-5. In silico: Network pharmacology Clinical trials: Human patients.	Inhibits SARS-CoV-2 infection and replication. Inhibits SARS-CoV-2’s 3-chymotrypsin-like protease. Inhibits papain-like protease. Targets IL-17, NF- κB, and TNF signaling pathways Lower hospitalization and pneumonia.	[118,232,233,234,235,236]
Naringenin	In vitro: Vero E6 cells In silico: Molecular docking; network pharmacology.	Inhibits SARS-CoV-2 infection Inhibits the interaction between spike glycoprotein and AC2 receptor Inhibits 3CLpro Decreases ACE receptor activity Activates *AKT1* gene Targets nsp3, nsp7, nsp8, and nsp12 Inhibits RNA polymerase and TPC2	[201,237,238,239,240]

Abbreviations: severe acute respiratory syndrome coronavirus 2 (SARS-CoV-2), protease 3 chymotrypsin-like protease (3CLpro), angiotensin-converting enzyme 2 (ACE2), toll-like receptor (TLR), hypoxia-inducible factor (HIF), vascular endothelial growth factor (VEGF), tumor necrosis factor (TNF), phosphodiesterase 4 (PDE4), main proteinase (MPro), alkaline phosphatase (ALP), C-reactive protein (CRP), lactate dehydrogenase (LDH), non-structural proteins (nsp), interleukin (IL), aspartate transferase (AST), T-Cells cluster of differentiation (TCD), Regulatory T (TReg), forkhead box P3 (FOXP3), transforming growth factor beta (TGF-β), papain-like protease (PLpro), transmembrane protease, serine 2 (TMPRSS2), RNA-dependent RNA polymerase (RdRp), tumor protein p53 (TP53), albumin (ALB), mitogen-activated protein kinase (MAPK), advanced glycation end products (AGE), receptor for advanced glycation end products (RAGE), epidermal growth factor receptor (EGFR), phosphatidylinositol 3-kinase (PI3K)/protein kinase B (AKT), long-interval intracortical inhibition (LICI), long-term potentiation (LTP), AKT Serine/Threonine Kinase 1 (*AKT1*), target two-pore channel 2 (TPC2).
